# Uncertainty around the Long-Term Implications of COVID-19

**DOI:** 10.3390/pathogens10101267

**Published:** 2021-10-01

**Authors:** Marc Desforges, Deepti Gurdasani, Adam Hamdy, Anthony J. Leonardi

**Affiliations:** 1 Centre Hospitalier Universitaire Ste-Justine and Faculté de Médecine, Département de Microbiologie, Infectiologie et Immunologie, Université de Montréal, Montreal, QC H3T 1J4, Canada; 2 Queen Mary University of London, London E1 4NS, UK; d.gurdasani@qmul.ac.uk; 3 Panres Pandemic Research, Newport TF10 8PG, UK; adam@panres.net; 4 Johns Hopkins Bloomberg School of Public Health, Johns Hopkins University, Baltimore, MD 21205, USA; aleona10@jh.edu

**Keywords:** SARS-CoV-2, COVID-19, long COVID, post-acute COVID-19 syndrome, reinfection, coronavirus, neuroinvasion

## Abstract

Severe acute respiratory syndrome coronavirus 2 (SARS-CoV-2) has infected more than 231 million people globally, with more than 4.7 million deaths recorded by the World Health Organization as of 26 September 2021. In response to the pandemic, some countries (New Zealand, Vietnam, Taiwan, South Korea and others) have pursued suppression strategies, so-called Zero COVID policies, to drive and maintain infection rates as close to zero as possible and respond aggressively to new cases. In comparison, European countries and North America have adopted mitigation strategies (of varying intensity and effectiveness) that aim primarily to prevent health systems from being overwhelmed. With recent advances in our understanding of SARS-CoV-2 and its biology, and the increasing recognition there is more to COVID-19 beyond the acute infection, we offer a perspective on some of the long-term risks of mutational escape, viral persistence, reinfection, immune dysregulation and neurological and multi-system complications (Long COVID).

## 1. Introduction

Severe acute respiratory syndrome coronavirus 2 (SARS-CoV-2) has infected more than 231 million people globally, with more than 4.7 million deaths recorded by the World Health Organization as of 26 September 2021 [1].

In response to the pandemic, some countries (New Zealand, Vietnam, Taiwan, South Korea and others) have pursued suppression strategies, so-called Zero COVID policies, to drive and maintain infection rates as close to zero as possible and respond aggressively to new cases [2]. In comparison, European countries and North America have adopted mitigation strategies (of varying intensity and effectiveness) that aim primarily to prevent health systems from being overwhelmed. These mitigation strategies are managed through social measures and public health surveillance systems. Alongside, they have focused on the development of therapeutics for managing acute infections, and vaccine development as a longer-term strategy for controlling the pandemic. At present, there are high levels of community transmission in several countries around the world, with daily cases in the tens of thousands [1]. Scientists concerned about this strategy have called for a pan-European strategy aimed at suppression [3].

With recent advances in our understanding of SARS-CoV-2 and its biology, and the increasing recognition there is more to COVID-19 beyond the acute infection [4], it is clear that there are several risks inherent in strategies that are unable to prevent surges in community transmission. We offer a perspective on these here and summarise the key findings in Table 1.

## 2. Viral Evolution

Vaccines rely on the induction of neutralizing antibodies to the virus. High levels of community transmission increase the risk of mutations that can escape the neutralizing antibodies, potentially rendering vaccines less effective [5,6,7]. Variant B.1.351 (now known as Beta), first identified in South Africa, showed evidence of escape from convalescent sera and potentially reduced vaccine effectiveness, and is already circulating in at least 32 countries [8]. Variant P1 (now known as Gamma) spread rapidly across Manaus at a point when an estimated 76% of the population was thought to have been exposed to SARS-CoV-2 during the first wave [9]. Variant Delta (B.1.617.2) is more transmissible and shows an 8-fold decrease in neutralization by vaccine sera [10], and reduced vaccine efficacy against symptomatic disease [11].

The ‘plasticity’ of the immunodominant domain of the viral Spike protein allows mutations to occur without disrupting ACE-2 receptor-binding, necessary for SARS-CoV-2 infection. This has important implications for mutational escape. This has been suggested for human coronavirus HCoV-229E [12]. The introduction of a mutated variant of the virus through the mink population in Denmark, reported to potentially be less sensitive to neutralizing antibodies, also raises the possibility of re-introduction of divergent strains through animal populations that could potentially pose a risk [13]. Vaccines are a major factor in bringing pandemics under control and require significant development time and investment. With a number of vaccines authorized and now in use around the world, every effort should be made to give vaccines the best chance of success. While vaccines may be amenable to updating, this will take time, and trialing across populations, during which many lives may continue to be lost. Even though coronaviruses like SARS-CoV-2 possess an RNA proofreading mechanism [14], the virus has proven to be adaptive, and allowing transmission at a level that increases the probability of mutational escape risks more efficient human-to-human transmission which may seriously undermine this strategy.

Immune escape is not the only long-term risk of continued evolution. Transmissibility and disease severity can increase as a result of evolution, and a study of the impact of variants of concern on disease severity concluded that there was a signal toward increased disease severity associated with Delta variant and a lower Ct value and longer viral shedding that could provide a mechanism for increased transmissibility [15]. Some researchers expect continued evolution will attenuate the virus over time, but even if this happens it is difficult to predict how long this process might take, and so far new variants of concern have corresponded with an increase in disease severity. Population immunity may blunt the impact of increased virulence and/or transmissibility, but when combined with a degree of immune escape or waning immunity, such evolution could prove problematic.

## 3. Viral or Antigenic Persistence

There is some evidence to suggest that human coronaviruses, like animal coronaviruses, may be capable of persistence in the host in a low replication state [16]. Persistent shedding of virus RNA in nasopharyngeal swabs and feces samples has been reported for up to 6 weeks among hospitalized patients [17]. A recent report suggests that SARS-CoV-2 particles and antigen may persist in the human gut [18] up to 4 months following infection, in the presence of neutralizing antibodies, even in individuals who were asymptomatic at the point of follow-up. Whether these represent viable viral particles is unclear. Expectedly, B cells in these individuals continued to evolve in a way that indicates continued antigen exposure [19]. Autopsy examinations have revealed persistence of viral RNA in lung pneumocytes, endothelial tissue and olfactory neuroepithelium [20] weeks after onset of symptoms, highlighting the potentially serious implications of viral persistence [21]. Reports of prolonged shedding of infectious virus for >70 days in an immunocompromised patient with evidence for continuous mutational evolution of virus in vivo suggests persistence of viable and replication-competent virus for long periods in certain circumstances [22]. Finally, a separate report claims persistent replication of SARS-CoV-2 for a median of 85–105 days after recovery for 5.3% of individuals in a cohort. Despite antibody responses comparable to the non-persistent RNA group, these individuals had CD8 T-cell responses of significantly greater magnitude, possibly indicating immunological compensation [23]. There is much we do not yet know about the prevalence, mechanisms, clinical implications and the long-term impact of antigenic or viral persistence with SARS-CoV-2.

## 4. Reinfection

To date, at least 362 genetically sequenced cases of re-infection with SARS-CoV-2 have been documented [24]. It is likely this is an underestimate given the high threshold of evidence required to confirm re-infection. In Québec there have been at least 1588 cases of presumed reinfection [25] and in England there have been at least 23,105 possible reinfections [26]. Even though reinfections by endemic human coronaviruses are possible and frequent [27], with a median interval of nine months [28], the mechanism of reinfection by human coronaviruses is poorly understood. It is unclear whether this is due to viral evolution and mutational escape, a declining immune response, or a combination of the above. Reinfection observed with SARS-CoV-2, has been shown to occur between 10–282 days following primary infection among documented cases [24], with some individuals experiencing a less or comparably severe illness the second time [29], and others a more severe one [30].

Some governments expect vaccines to enable future infections or reinfections to act as immune boosters [31], but there is evidence that even after multiple infections and full vaccination, reinfection can result in more severe disease [32]. A study of US healthcare workers found that hospitalization was more likely following reinfection [33]. The UK SIREN study suggested that although prior infection provided ~80% protection against reinfection at 5 months, compared with controls, symptomatic re-infections were still seen with high virus loads and potential for transmission. While the incidence of re-infection remains unknown, the potential for reinfection at short intervals, especially with more severe subsequent infections, presents a serious risk with high levels of community transmission [34]. This aspect is even more important now, given the potential ability of new virus variants in many parts of the world to at least partially escape the immune response directed at the wild-type virus or previous variants [8].

## 5. Immune Dysregulation

SARS-CoV-2 causes profound immune dysregulation during acute infection [35]. Functional exhaustion of antiviral lymphocytes has been reported [36], alongside the production of cytokines akin to “a polyclonal, superantigen-driven T-cell activation” [37]. A superantigen-like region on the spike protein may drive responses that manifest as a Multisystem Inflammatory Syndrome in children (MIS-C) [38].

SARS-CoV-2 has several important mechanisms of immune escape, that may influence disease severity, and favor antigenic/viral persistence. The ORF8 and NSP1 viral proteins are able to reduce MHC I expression, and shut down host protein translation, disarming key components in the adaptive and innate immune response [39,40,41], permitting antigen to accumulate and spread [42]. There is evidence that SARS-CoV-2 can infect leukocytes [43], including T lymphocytes, in which the virus may use the CD4 molecule, facilitating further evasion of the immune response [44]. Early work on the new variants suggests the N501Y mutation may facilitate immune escape by weakening cooperation between T and B cells [45].

Peripheral lymphopenia, leukopenia, and thrombocytopenia are key features of the clinical syndrome associated with COVID-19 among hospitalized patients [46]. The potential impact of SARS-CoV-2 on hematopoietic stem cells is also a cause for concern. Exposure to the viral spike antigen alone in vitro has been shown to cause functional and expansion deficits in hematopoietic stem cells and progenitor cells [47]. Similar effects occurring in patients with continued antigenic exposure, could manifest as bone marrow failure [48]. Furthermore, there is evidence of aberrant CD4+ and CD8+ responses that may continue for longer periods following acute infection [49]. Even after ten weeks of COVID-19 infection, cytotoxic CD3, CD4, and CD8 T cells remain activated in convalescent patients [50]. Patients with mild infection exhibit increased exhaustion gene signatures in CD8+ T cells [51]. Some patients develop autoimmune responses and clinically identifiable auto-reactivity in severe SARS-CoV-2 infection [52]. These include a Kawasaki-like disease in children, anti-cardiolipin antibody related thrombotic events, auto-immune hemolytic anemia and thrombocytopenia [53]. Broad auto-reactivity has been identified among those infected with SARS-CoV-2, associated with impaired virological control through inhibition of immunoreceptor signaling. This auto-reactivity has been shown to correlate with the severity of clinical outcomes [21]. There is also evidence SARS-CoV-2 can subvert CD8+ T cell surveillance through escape mutations in MHCI-restricted viral epitopes [54,55]. The longer-term impacts on the immune system are yet unknown, but particularly in light of possible viral/antigenic persistence and the potential for reinfection, we should be concerned about the risk of lasting immune dysfunction and damage. There has even been a suggestion acute COVID-19 disease should not be regarded as viral pneumonia, but instead considered an autoimmune disease [56]. SARS-CoV-2 has also been shown to play a pivotal role in inducing the autoimmune hyperthyroidism of Graves’ disease [57]. We echo the British Society for Immunology’s call for urgent research in this area [58].

## 6. Neurological and Multi-System Complications (Long COVID)

COVID-19 is a multi-system disease with the potential for long-term complications. Long COVID is a catchall term conferred by patient advocacy groups to cover a variety of symptoms [59]. Work is underway to understand the diversity and causes of symptom presentation, but for our purposes, we will use Long COVID as a term to refer to post-acute symptoms.

Data from the UK Office for National Statistics (ONS) suggest that 11.7% of all those infected report symptoms lasting for >12 weeks [60], with 12.3% of secondary school pupils reporting symptoms for more than 4 weeks [61]. The REACT-2 survey estimates more than 2 million people in the UK have suffered from Long COVID symptoms persisting for 12 weeks or more [62], with ONS estimating 1 million people in the UK currently have long COVID, of whom 2/3rds have some limitation in day to day activities, and ~384,000 have had symptoms for over 1 year [63].

Although COVID-19 has been described as a respiratory syndrome, evidence supports the involvement of multiple organ systems, with fibrosis, and inflammation in the lung, heart, kidneys, central nervous system (CNS), liver, adrenal glands, bone marrow, lymph nodes and gastrointestinal tract [64]. SARS-CoV-2 infection has also been associated with serious thrombotic complications, including strokes, pulmonary embolism, and cardiac injury [65,66]. Case reports of acute parkinsonism following infection by SARS-CoV-2 [67] and growing evidence of increased risk of neuro-psychiatric disease up to 6 months following acute infection [68,69,70] suggest the potential for serious long-term complications with COVID-19. The extent to which these effects are a result of direct infection versus the impact of the inflammatory response to the virus is unclear. Natural SARS-CoV-2 neuroinvasion has been evidenced by autopsy [21,71,72] and an imaging study of hundreds of COVID-19 patients scanned before and after infection showed evidence of loss of grey matter in regions of the brain associated with smell, memory, and emotional processing following infection when compared to matched controls [73]. A study of severe COVID-19 patients, comparing neurological manifestations to other diseases, including terminal influenza, found dysregulation of brain and choroid plexus cell types and COVID-diseases associated microglia and astrocyte subpopulations that share features with pathological cell states reported in human neurodegenerative disease [71]. Notably, T cells infiltrated the brain Parenchyma in COVID-19 patients while no such infiltration was observed in lethal cases of Flu [74]. A study of pediatric patients found that despite lower initial severity at the acute stage of the infection, pediatric patients demonstrated on average 5 months later a similar brain hypometabolic pattern as that found in adult Long COVID patients, involving bilateral medial temporal lobes, brainstem, cerebellum and the right olfactory gyrus [75]. Cognitive decline following mild and severe COVID-19 infection has been evidenced in a recent study and we agree with the authors call for further research with longitudinal and neuroimaging cohorts to plot recovery trajectories and identify the biological basis of cognitive deficits in COVID-19 survivors [76].

Long-term follow-up studies of those with COVID-19 infection show that symptoms of breathlessness, as well as lung changes on imaging can persist in large numbers of patients beyond 12 weeks. These long-term effects are consistent with those observed in SARS-CoV-1, where 4.6% still had visible lesions on their lungs, and 38% had reduced diffusion capacity after 15 years following acute infection [4]. In recent studies >40% of COVID-19 patients report breathlessness, and >50% fatigue even 2 months after hospitalization, and 52% of home isolated young adults experienced Long COVID symptoms at 6 months following COVID-19 infection [77].

## 7. Conclusions

The above findings highlight that with COVID-19 we are not dealing solely with an acute infection with short-term clinical risks. There is the potential for significant long-term health implications and while there remains much to be understood, the precautionary principle would advocate for taking approaches that minimize this potential risk. To this end public health policy should focus on significantly reducing community transmission alongside vaccine roll-out. Focusing on hospitalizations and deaths as the only outcomes is short-sighted. Relying on post-vaccine infection as an immune ‘booster’ may carry serious risk as such infections can result in increased disease severity [32].

It is essential to reduce long-term risks from this novel virus until we understand them better, as well as reduce the risk of new variants emerging by suppressing transmission. There is now extensive evidence that transmission can be reduced through widespread use of face masks [78,79,80,81], better ventilation [82] and air filtration [83]. Contact tracing and isolation are established and effective methods of reducing transmission. Governments should implement these mitigation measures in order to protect long-term public health and the effectiveness of vaccines.

**Table 1:** Key Findings.

Finding	Ref.
Beta/B.1.351 is markedly more resistant to vaccine and convalescent sera than original wild type.	Wang et al. (2021) [8]
The emergence of the P1 variant saw continued transmission despite a 66% attack rate in June 2020, rising to 76% in October 2020.	Buss et al. (2020) [9]
Delta is more transmissible, has partial sera escape, and reduces vaccine efficacy.	Mlcochova et al. (2021) [10] Lopez et al. (2021) [11]
Viral evolution can result in increased disease severity.	Ong et al. (2021) [15]
SARS-CoV-2 particles and antigen may persist in the human gut up to 4 months.	Gaebler et al. (2021) [18]
Immunocompromised patients can facilitate rapid evolution and shed for longer than 70 days.	Avanzato et al. (2020) [22]
SARS-CoV-2 reinfections have been more mild and more severe.	Parry (2020) [29] Tillett et al. (2020) [30]
Even following vaccination, reinfections can be more severe.	Jayanthi et al. (2021) [32] Slezak et al. (2021) [33]
Symptomatic reinfections have potential for onward transmission.	Hall et al. (2020) [34]
SARS-CoV-2 causes immune dysregulation and functional exhaustion of lymphocytes.	Kalfaoglu et al. (2020) [35] Zheng et al. (2020) [36] Kratzer et al. (2020) [50]
SARS-CoV-2 contains a superantigen that can manifest in MIS-C.	De Biasi et al. (2020) [37] Cheng et al. (2020) [38]
SARS-CoV-2 suppresses adaptive and innate immunity using a variety of mechanisms.	Park (2020) [39] Zhang et al. (2020) [40] Thoms et al. (2020) [41]
SARS-CoV-2 manifests in lymphopenia, leukopenia, and thrombocytopenia.	Guan et al. (2020) [46]
Spike protein causes hematopoietic functional deficits in vitro.	Xu et al. (2020) [47]
Cytotoxic T cells remain activated in the convalescent.	Chen et al. (2020) [49] Kratzer et al. (2020) [50]
SARS-CoV-2 causes autoimmunity.	Bussani et al. (2020) [19] Woodruff et al. (2020) [52] Talotta & Robertson (2020) [53] Zuniga et al. (2021) [56] Murugan & Alzahrani (2020) [57]
SARS-CoV-2 has exhibited potential for T cell epitope escape.	Agerer et al. (2020) [54] Pretti et al. (2020) [55]
SARS-CoV-2 infection causes lasting symptoms.	Whitaker et al. (2021) [61] Blomberg et al. (2021) [62]
SARS-CoV-2 infection causes strokes, pulmonary embolism, and cardiac injury.	Merrill et al. (2020) [64] Bose & McCarthy (2020) [65]
SARS-CoV-2 is neuroinvasive.	Matschke et al. (2020) [21] Geidy et al. (2021) [70] Song et al. (2021) [71]

## Data Availability

Not applicable.
